# Initial experiences and technical insights of pulmonary vein isolation with FARAPULSE pulsed field ablation in patients implanted with WATCHMAN left atrial appendage closure devices: The first report in Japan

**DOI:** 10.1002/joa3.70065

**Published:** 2025-04-15

**Authors:** Ryuki Chatani, Hiroshi Tasaka, Shunsuke Kubo, Mitsuru Yoshino, Kazushige Kadota

**Affiliations:** ^1^ Department of Cardiovascular Medicine Kurashiki Central Hospital Kurashiki Japan

**Keywords:** atrial fibrillation, FARAPULSE, intracardiac echocardiography, left atrial appendage closure, pulsed field ablation

## Abstract

We performed pulsed field ablation using FARAPULSE after left atrial appendage closure (LAAC). We should confirm flower configurations did not overlap with the LAAC device using intracardiac echocardiography and 3‐D mapping system and without LAAC device artifacts by real‐time electrograms. If overlapping, push the flower configuration deeper and tilt posteriorly to resolve.

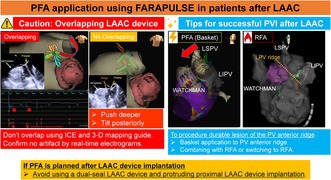

## 1 INTRODUCTION

The feasibility and safety of atrial fibrillation (AF) ablation through thermal ablation in the presence of a percutaneous left atrial appendage closure (LAAC) device have been reported.^1^ However, the feasibility and safety of AF ablation through a novel non‐thermal pulsed field ablation (PFA) in the presence of an LAAC device remain unclear. Here, we report the feasibility of AF ablation using the FARAPULSE PFA system (Boston Scientific) in the presence of an LAAC device through a case series.

## 2 DESCRIPTION

### 2.1 Case 1

**FIGURE 1:**
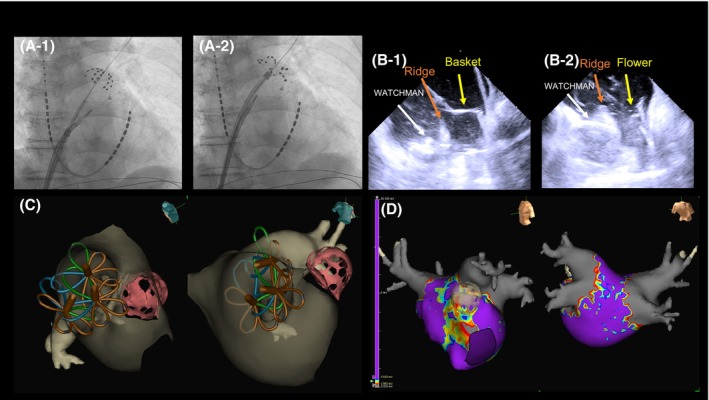
(A) Fluoroscopic image (A‐1: Basket; A‐2: Flower) (B) ICE showing no overlap between WATCHMAN and FARAPULSE (B‐1: Basket; B‐2: Flower). (C) Flower configuration application shadows during LSPV isolation. (D) Post‐PFA map.

An 84‐year‐old man with a high bleeding risk and a history of ischemic stroke was hospitalized due to heart failure as a result of persistent AF. He had undergone WATCHMAN FLX 27‐mm device implantation 24 months prior to the index procedure. Here, we performed pulmonary vein isolation (PVI) using the FARAPULSE PFA system. In this case series, intracardiac echocardiography (ICE) was performed before and after the PFA procedure to confirm the absence of device leakage or thrombi on the surface around the WATCHMAN device in the left atrium. During PFA application to the left superior pulmonary vein (LSPV), we confirmed that the flower configuration did not overlap with the LAAC device using ICE and a 3D‐mapping system guide (Figure 1). Post‐validation mapping was performed, which showed that all four pulmonary veins were electrically isolated. No complications were observed. The patient continued the original anticoagulation therapy (edoxaban: 15 mg daily) after discharge.

### 2.2 Case 2

**FIGURE 2:**
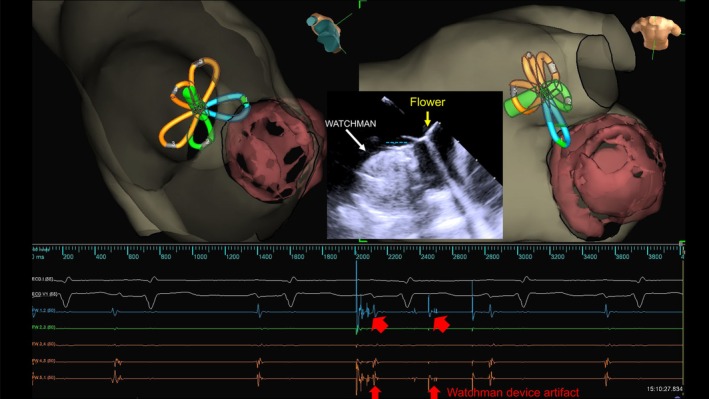
FARAPULSE with the flower configuration overlapping on the WATCHMAN device (blue dotted line). The artifacts (red arrow) were recorded using the FARAWAVE electrodes encountering the adjacent WATCHMAN device.

**FIGURE 3:**
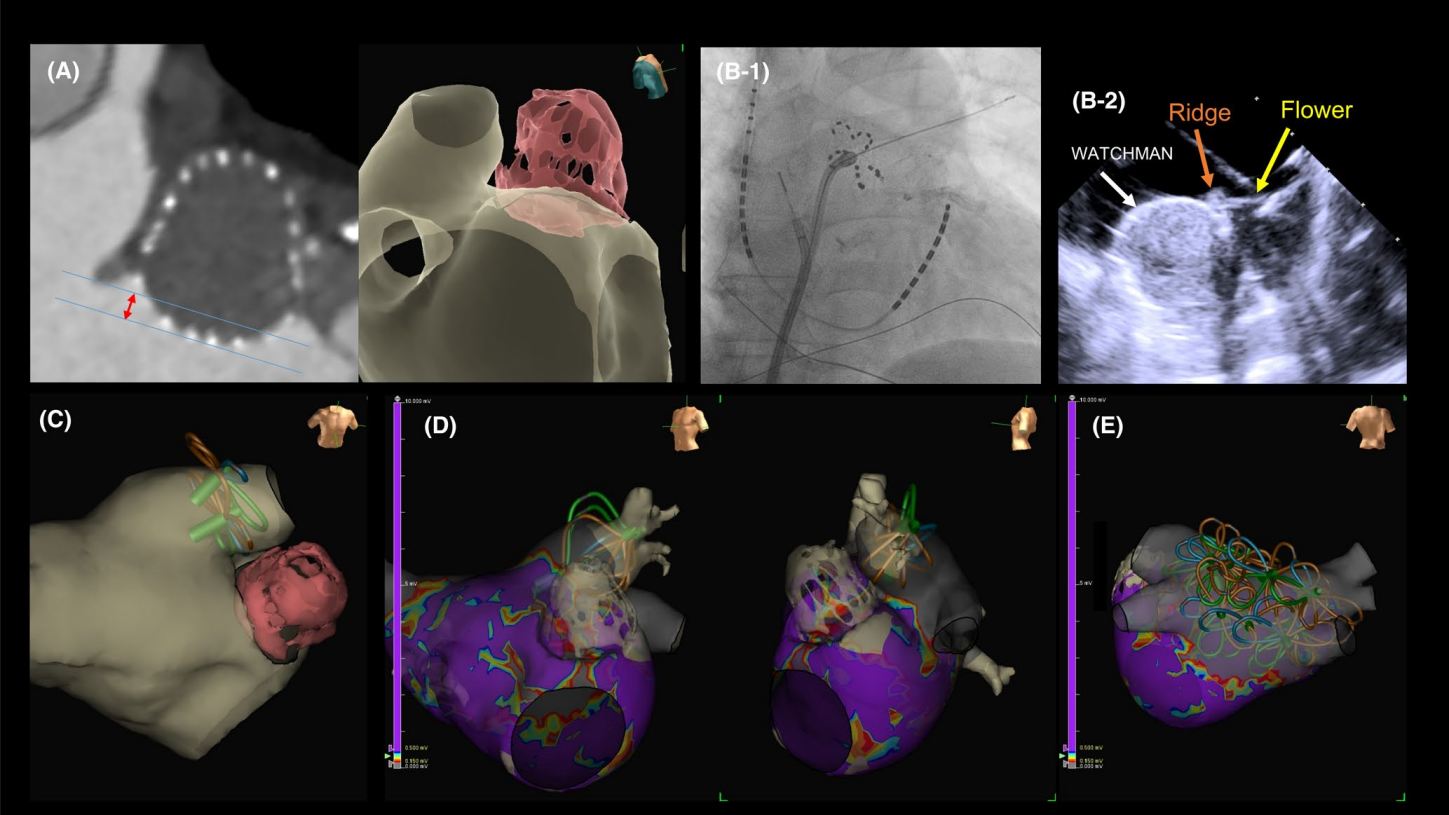
(A) Computed tomography image between LSPV ridge and WATCHMAN (4.4 mm). (B) Image showing no overlap between WATCHMAN and FARAPULSE (B‐1: Fluoroscopic image; B‐2: ICE). (C) Flower configuration application shadows during LSPV isolation. (D) Basket configuration application shadows during LSPV isolation and post‐PFA map. (E) Final post‐PFA map.

A 58‐year‐old man with heart failure with reduced ejection fraction who was undergoing dialysis had a history of LAA thrombus and had a high bleeding risk. He had undergone WATCHMAN FLX 31‐mm device implantation 15 months prior to the index procedure. We performed AF ablation using the FARAPULSE PFA system. Cardiac computed tomography revealed that the top of the LAAC device protruded 4.4 mm from the LSPV ridge at ablation. Interestingly, in this case, real‐time electrograms revealed LAAC device artifacts recorded by the FARAWAVE electrodes, which encountered the device during antral PFA with the flower configurations (Figure 2). We resolved this interference by pushing a little deeper and more posteriorly. Furthermore, because this was a long‐standing persistent AF, we decided to also isolate the posterior wall. A post‐validation map demonstrated that all four pulmonary veins and the posterior wall were electrically isolated (Figure 3). No complications were observed. The patient continued the original anticoagulation therapy (warfarin) for 3 months.

### 2.3 Case 3

A 76‐year‐old man with a history of major bleeding had undergone WATCHMAN FLX Pro 27‐mm device implantation 3 months prior to the index procedure. We performed AF ablation using the FARAPULSE PFA system to address his frequent palpitations after LAAC. During PFA application to the LSPV, we confirmed that no overlapping occurred with the LAAC device using only the 3D‐mapping system; the usage of ICE for this purpose was difficult as there was no steerable sheath in this case. The distance between WATCHMAN and FARAPULSE was 8 mm. A post‐validation map demonstrated that all four pulmonary veins were electrically isolated. No complications were observed. The patient continued the original anticoagulation therapy (edoxaban: 15 mg daily) for 3 months.

The patient data are summarized in Table 1.

**TABLE 1:** Patient characteristics.

	Case 1	Case 2	Case 3
Age	84 years	58 years	76 years
Sex	Male	Male	Male
The period from LAAC to CA	24 months	15 months	3 months
CHADS_2_ score	4	3	2
CHA_2_DS_2_‐VASc score	5	4	3
HAS‐BLED score	4	3	3
Type of atrial fibrillation	Persistent	Long standing	Paroxysmal
Left ventricular ejection fraction	60%	35%	61%
Left atrial diameter	44 mm	51 mm	37 mm
Left atrial volume	96 mL	141 mL	74 mL
Left atrial volume index	55 mL/cm^2^	89 mL/cm^2^	46 mL/cm^2^
WATCHMAN device type	FLX	FLX	FLX Pro
LAAC device size	27 mm	31 mm	27 mm
The protrusion of LAAC device	None	4.4 mm	None
CA procedure time	113 min	151 min	96 min
Rhythm at 3 months	Sinus	Sinus	Sinus

Abbreviations: CA, catheter ablation; LAAC, percutaneous left atrial appendage closure.

## 3 DISCUSSION

Theoretically, catheter–device contact and the delivery of energy on the LAAC device surface pose risks of various complications, such as device dislodgment, device‐related thrombosis (DRT), and peri‐device leakage (PDL). In this report, PFA was safely performed after WATCHMAN device implantation.

### 3.1 Appropriate timing of PFA after LAAC

Although there is no established protocol for the optimal waiting time between LAAC and subsequent catheter ablation, it is reasonable to wait for at least 6 months in the case of radio frequency ablation (RFA) for reducing complications.^2^ However, we have reported that RFA using CARTOSOUND (Biosense‐Webster) as a supplement in the presence of an implanted LAAC device was safe and feasible, even in the early phase (≤6 months).^1^ Recent research has shown that the interruption of PFA application is not related to the time of LAAC endothelization (derived from the time from LAAC implantation to PVI).^3^ Theoretically, PFA energy is less affected by the LAAC device than RFA, and PFA after LAAC described in this report is feasible even in the early phase (3 months). The accumulation of more early phase cases is needed to validate this hypothesis.

### 3.2 Technical issues of PFA after LAAC device implantation

In the treatment of the cases described in this report, catheter ablation was performed with the FARAPULSE system as a PFA device, which has a unique safety mechanism based on confirmation of nominal impedance between ablative electrodes. Currently, the FARAPULSE system is the only available PFA device for patients implanted with an LAAC device in Japan.

In addition, WATCHMAN devices were used for LAAC in this case series. If an Amplatzer Amulet device was used, the device disk may have overlapped the LSPV ridge, preventing PFA application due to interference from the LAAC device.^4^ In case 2, the device was large and implanted proximally, causing artifacts between the LAAC device and the PFA electrodes; this interference occurred even when the WATCHMAN device was used. Fortunately, this interference was resolved by pushing the flower configurations a little deeper and tilting them more posteriorly. However, this approach may result in non‐durable lesion with reconnection of the left anterior PV ridge. Therefore, the firm PFA application with the basket configuration to the anterior PV ridge during LSPV isolation is important. Additionally, for successful PVI, switching to or combining with point‐by‐point RFA ablation is an option. If PFA is planned after LAAC, avoid using an Amplatzer Amulet device and protruding proximal implantation. In patients with a protruded LAAC device, a combination of PFA and RFA may be promising.

### 3.3 Prospects of PFA after LAAC

In patients eligible for ablation therapy, it is reasonable to perform ablation therapy before LAAC because medical resources are limited. The advantage of performing LAAC first is that it prevents periprocedural complications such as stroke and allows for interruption of oral anticoagulants due to periprocedural major bleeding; the disadvantage is the risk of device complications. Given the lack of reports of endothelial damage from PFA, DRT or PDL due to thermal degeneration is less likely. The advantages of PFA, such as short procedure time and absence of char or coagulum, favor its use in patients implanted with an LAAC device. In the future, it may become common practice to perform PFA and LAAC simultaneously in selected patients.^5^

## FUNDING INFORMATION

N/A.

## CONFLICT OF INTEREST STATEMENT

Dr. Chatani and Dr. Kubo are clinical proctors for Boston Scientific and have received honoraria from Boston Scientific. All other authors declare no conflicts of interest.

## ETHICS APPROVAL STATEMENT

This study was approved by the Institutional Review Board of Kurashiki Central Hospital (approval no. 4614).

## PATIENT CONSENT STATEMENT

Written informed consent was obtained from the patient for the publication of this report.

## CLINICAL TRIAL REGISTRATION

N/A.
